# Supplementary material for: A high-resolution mRNA expression time course of embryonic development in zebrafish
Source: eLife. 2017 Nov 16;6:e30860. doi: 10.7554/eLife.30860 (PMC5690287; doi:10.7554/eLife.30860)
Supplement: Supplementary file 6. [file elife-30860-supp6.zip › biolayout-clusters-files/Cluster203-genes.html]

Cluster203


# Cluster203: Genes

| Ensembl ID | Gene Name | Chr | Start | End | Biotype |
| --- | --- | --- | --- | --- | --- |
| ENSDARG00000076416 | SHROOM2 | 6 | 30385401 | 30466504 | protein\_coding |
| ENSDARG00000024654 | pgm3 | 4 | 5776400 | 5786739 | protein\_coding |
| ENSDARG00000038563 | pitrm1 | 2 | 48234893 | 48265245 | protein\_coding |
| ENSDARG00000079915 | pot1 | 25 | 26962599 | 27048692 | protein\_coding |
| ENSDARG00000017500 | rock2a | 13 | 32684791 | 32768898 | protein\_coding |
| ENSDARG00000079900 | shroom4 | 7 | 24458401 | 24541867 | protein\_coding |
| ENSDARG00000069175 | vps4b | 2 | 13573148 | 13651165 | protein\_coding |
